# Transposable element subfamily annotation has a reproducibility problem

**DOI:** 10.1186/s13100-021-00232-4

**Published:** 2021-01-23

**Authors:** Kaitlin M. Carey, Gilia Patterson, Travis J. Wheeler

**Affiliations:** 1 Department of Computer Science, University of Montana, 32 Campus Drive, Missoula, MT USA; 2 Institute of Ecology and Evolution, University of Oregon, 272 Onyx Bridge, Eugene, OR USA

**Keywords:** Transposable elements, Interspersed repeats, Subfamilies, Segmental duplications

## Abstract

**Background:**

Transposable element (TE) sequences are classified into families based on the reconstructed history of replication, and into subfamilies based on more fine-grained features that are often intended to capture family history. We evaluate the reliability of annotation with common subfamilies by assessing the extent to which subfamily annotation is reproducible in replicate copies created by segmental duplications in the human genome, and in homologous copies shared by human and chimpanzee.

**Results:**

We find that standard methods annotate over 10% of replicates as belonging to different subfamilies, despite the fact that they are expected to be annotated as belonging to the same subfamily. Point mutations and homologous recombination appear to be responsible for some of this discordant annotation (particularly in the young Alu family), but are unlikely to fully explain the annotation unreliability.

**Conclusions:**

The surprisingly high level of disagreement in subfamily annotation of homologous sequences highlights a need for further research into definition of TE subfamilies, methods for representing subfamily annotation confidence of TE instances, and approaches to better utilizing such nuanced annotation data in downstream analysis.

## Introduction

Transposable elements (TEs) are usually annotated within a genome using a tool, such as RepeatMasker [1], that compares a genome to a library of known TEs, such as Repbase [2]. In such a library, TE remnants are classified into families and subfamilies. Subfamilies are in some cases included in a TE library in order to increase annotation coverage, but they more often represent the history of replication and divergence of a family. This history can be complex, with numerous replication bursts leading to clusters of related TEs [3, 4]. Standard practice is to reconstruct and define such subfamilies based on shared diagnostic sequence variation [5, 6] within such bursts. Because annotation with these subfamilies is believed to give some indication of a sequence’s historical context, it is important that such annotation be reproducible.

### Adjudication of subfamily annotation candidates

When annotating TE instances within a genome, the common strategy is to use sequence alignment software to compare genomic sequence to each (sub)family in the TE library. When a collection of TE elements within this database are similar to each other, they will all tend to align well to the same genomic sequence, so that one genomic region may attract many competing annotations. The common strategy for selecting which annotation is preferred (a process that we call *adjudication*) is to select a single highest-scoring alignment.

### Annotation reliability

We define a subfamily as being reliably annotated if nearly all instances of the subfamily that were inserted in some past time period will be annotated as belonging to that subfamily in the annotation of extant genomic sequence. In this study, we evaluate the reliability of subfamily annotation, focusing attention on the two families with the largest distribution of subfamilies found in the human genome: Alu and L1. Alus are young and short, and carry a significant risk of cross-annotation due to straightforward mechanisms such as random point mutation and gene conversion; L1s are older, longer elements with complex histories, and discordance is likely due to more complex mechanisms such as recombination and incomplete cataloging of subfamilies.

### Biological replicates to assess subfamily annotation reliability

Because we do not know the actual history of extant TE instances in the genome, we evaluate the reliability of subfamily annotations using biological replicates - pairs of TE instances that are descended from a single TE insertion, differentiated only by mutations accumulated independently and randomly since the two sequences split from their shared ancestor. If subfamily annotation is reliable, both copies should be annotated as belonging to the same subfamily. We consider two sources of biological replicates (segmental duplication and species divergence) and demonstrate that >10% of replicate Alu and L1 pairs are classified into different subfamilies.

The intention of this analysis is not to exhaustively enumerate reliability measures across all families, library composition, annotation software, or parameterizations; surely the precise extent of annotation reliability would vary with specifics of such a survey. Even so, we believe that these results highlight that reasonable concerns exist regarding the reliability of subfamily assignment during genome annotation. We hope that this observation, and mechanisms for quantifying annotation reliability and uncertainty, will motivate future work in identifying rigorous and effective measures for improving and accounting for reliability.

In the sections that follow, we describe experiments that quantify the extent of annotation (un)reliability, explore potential sources of discordant annotation, and discuss a new mechanism for roughly estimating reliability.

## Results

### TE subfamily annotation shows high level of discordance in biological replicates

To understand the reliability of subfamily annotation, we have analyzed two datasets that serve as biological replicates: duplicates found in the human genome due to segmental duplication, and duplicates shared by humans and chimp, due to speciation. We call pairs that are classified into different subfamilies despite being derived from a common TE insertion event *discordant annotation*, and find that more than 10% of TE pairs are discordantly annotated in both datasets.

#### TE annotation discordance in segmental duplications

**Fig. 1:**
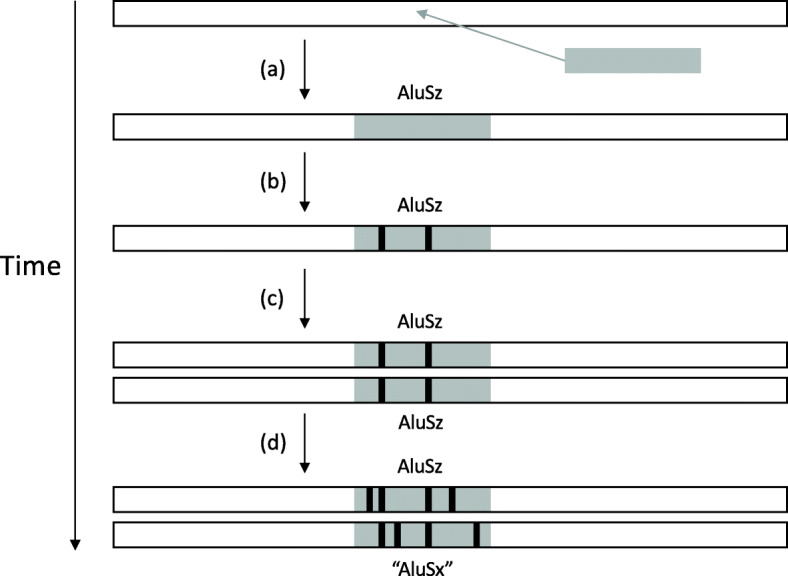
Segmental duplications generate replicate, divergent TE copies. **a** A TE (grey rectangle) is integrated into a segment of an ancestral genome (white rectangle). The inserted element belongs to the subfamily AluSz. **b** Over time, this TE instance accumulates random mutations (black bars), diverging from the original inserted sequence. **c** A segment around the TE is duplicated, resulting in two identical copies of the TE instance (and surrounding region). **d** Over time, each TE copy accumulates random mutations, so that they are no longer identical. In this imaginary situation, the newly accumulated mutations are sufficient to cause the bottom copy to be identified as belonging to subfamily AluSx

One source of biological replicates is segmental duplication [7], in which long (>1000 base pair) regions of DNA have been duplicated one or more times. When a TE is present within a region that is duplicated, the TE instance in the original segment and the instance in the duplicate segment are biological replicates (Fig. 1).

We identified all instances where a TE was copied as part of a segmental duplication, restricting our analysis to segments that duplicated only once. In order to ensure that evaluated pairs are homologous, and not accidentally paired during segmental duplicate alignment, we excluded instances in which one TE remnant was less than 50 base pairs long, a TE in one segment overlapped multiple TEs in the other segment, or a TE in one segment overlapped less than 80% of a TE in the other segment. There were 16,962 instances of these straightforward TE duplications. A pair was labeled as discordant if RepeatMasker’s annotation placed the TEs into different subfamilies. The extent of discordant annotation varied among families (Table 1), but was high in both younger TE families (Alu, 12.4%) and older families (L1, 14.1%). This table focuses on Alu and L1 because these are families for which subfamilies are intended to provide some insight into biology; in these cases, it is particularly important that subfamily classification be reliable. Some other families (e.g. MIR, L2, MLT in human) may consist of subfamilies created simply to improve annotation sensitivity; these subfamilies also demonstrate high discordant annotation within our segmental duplication analysis (e.g. MIR=10.4%, L2=15.7%, and MLT=6.7%).

**Table 1:** Rate of discordant subfamily annotation in human segmental duplications

Family	# Subfamilies	# in genome	# pairs in filtered seg. duplications	# discordant	% discordant
Alu	47	1196725	10347	1290	12.4
L1	131	951429	6615	933	14.1

TE pairs within segmental duplications were identified as described in the text (hg19 segmental duplicates from [8], TEs based on RepearMasker+Repbase annotation on hg19, filtered for length and quality of overlap between segmental duplicates). Among these, TEs belonging to the Alu and L1 families were considered, because these subfamilies are intended to represent biological history. Discordant annotations are those in which one element in a TE pair is assigned to one subfamily in Repeatmasker, while the other element in the pair is assigned to a different subfamily

Table 2 shows pair-annotation relationships for the three main types of Alu subfamilies: AluJ, AluS, and AluY. In all cases, the large majority of pairs are annotated with matching subfamilies; but when discordant annotation is observed, it is common that the mis-matched pair crosses from one subfamily type to another.

**Table 2:** Annotation of TE pairs in segmental duplications, for the three types of Alu subfamilies

	Concordant	Non-match	Non-match	Non-match	Other, e.g.	Mismatch
		AluJ	AluS	AluY	FRAM/FLAM	percent
AluJ	2308	254	69	10	134	16.8%
AluS	5776	69	629	77	24	12.2%
AluY	973	10	77	89	4	15.5%

In this table, each cell tallies the number of cases where one of the elements of a segmental duplicate TE pair belongs to the type specified by the row (from among AluJ, AluS, and AluY), and the other element belongs to type specified by the column. The first column captures concordant pairs (both entries share the same subfamily). In the next three columns, a cell captures the count of cases where one element belongs to one subfamily (specified by row), and the other element belongs to a different subfamily, and belongs to the type specified by the column (e.g. there are 254 cases in which an AluJ type instance is paired with another instance with a different subfamily that is still of the AluJ type; meanwhile there are 69 cases in which an AluJ type is paired with an AluS type). Mismatch percents (final column) exceed those in the previous table, because each discordant pair is double counted. The table is intended to highlight the differences in between-type discordance rates

#### TE annotation discordance in TEs shared by humans and chimps

We also considered biological replicates produced by species divergence, in which a single TE present in the common ancestor of human and chimpanzee yields two instances that have diverged along independent lines since a shared common ancestor. The pair of homologous TEs should be identified as belonging to the same subfamily. We correlated RepeatMasker annotation of TEs with homologous segments in the whole genome alignment of human (hg38) and chimp (panTro4) from the UCSC Genome Browser [9], using the UCSC liftOver tool. The rates of discordant annotation in homologous pairs of Alu and L1 TEs, summarized in Table 3, are similar to the rates in segmental duplications.

**Table 3:** Subfamily counts and rates of discordant annotation based on homologous TEs in humans and chimps

Family	# homologous pairs	% discordant
Alu	1093387	14.95%
L1	1050856	17.60%

RepeatMasker annotions of the hg38 human genome and panTro4 chimp genomes were paired using the UCSC liftover tool. Discordant annotation was identified as that in which lifted-over annotations differed at the subfamily level

### Drift via point mutations may explain some discordance in younger subfamilies

In cases where two TE subfamilies are distinguished by only a small number of diagnostic nucleotide substitutions, it is possible that a TE instance belonging to one subfamily will accumulate random point mutations at those diagnostic sites, leading to a change in annotated subfamily. Specifically, if half of the diagnostic sites switch from agreeing with the consensus for one subfamily to agreeing with the consensus for a single other subfamily, annotation may shift.

We quantified the expected frequency of such random subfamily drift, using a simple point mutation model. The model assumes that, after initial insertion of a TE instance in the genome (Fig 1b), the probability of a diagnostic site mutating away from the subfamily’s diagnostic nucleotide is simply the observed percent divergence between the subfamily consensus and individual instances of that subfamily; further, assuming a mutation occurs, the model assumes all resulting nucleotides are equally likely. We focused on Alu families, since these are young and the most apt to endure subfamily adjustment due to point mutations of diagnostic sites; see Methods for details. Probability of subfamily change due to drift was computed for each subfamily drifting to each other subfamily, and a weighted average was computed for each of AluJ, AluS, and AluY, based on the expected frequency of initial subfamily membership.

The results in Table 4 show that ∼7−8*%* of inserted AluS and AluY subfamily instances are expected to mutate such that they agree with other Alu subfamilies, i.e. are expected to produce discordant annotation; these may explain $\sim \frac {1}{2}$ of observed discordance. These changes are essentially always expected to occur within-type, so do not explain between-type changes (e.g. from AluY type to AluS type). The large majority of changes are due to (i) promiscuous interchange within a small clique of nearly-identical AluS subfamilies (AluSg, AluSz, AluSx, AluSx1, and AluSx3) and (ii) within another small AluY clique (AluYc, AluYf1, and AluYm1, and the AluY subfamily). Older AluJ subfamilies appear to be unlikely to convert due to point mutations. The subfamiles in these cliques are also responsible for much of the observed discordance.

**Table 4:** Expected conversion between Alu subfamiles based on a simple model of substitution mutations

	Non-match	Non-match	Non-match	Combined
	AluJ (%)	AluS (%)	AluY (%)	mismatch
AluJ	0.72	2e-5	8e-10	0.73%
AluS	2e-3	7.76	4e-3	7.77%
AluY	3e-5	0.02	6.69	6.71%

For each pair of subfamilies, we computed the probability of switching from one subfamily to another, based on the probability of changing the necessary number of diagnostic sites. Subfamily pairs were clustered by type, capturing the probability of converting from one of the types either within type (diagonal) or between types (off-diagonal). The final column is the sum of all probabilities of converting from the row header to any other subfamily

### The possible role of homologous recombination in discordant annotation

A complicating factor in counting discordant annotations is that TEs are hot spots for non-allelic homologous recombination [10, 11], due to the presence of many highly similar cousin sequences belonging to either the same or similar subfamilies. A common scenario is that a double-stranded break in one chromosome (the acceptor) is repaired using a similar sequence from another location (the donor) as a template [12, 13]. If the break occurs in one TE of a replicate pair, the donor sequence may be one of many cousin TE instances, possibly one from a different related subfamily. In this case the annotation system would be correct in assigning the pair of TEs to different subfamilies.

In the case of segmental duplication, the alignment of the segments surrounding paired TE instances will show some divergence since the duplication event, and it is expected that percent identity should be fairly consistent across the entire segment. After a recombination event, the whole-segment alignment is expected to show a reduced pairwise identity at the recombined region relative to the surrounding segmental duplication.

To gain some insight into the frequency of recombination, we applied a simple test to identify these significant dips in percent identity. We computed the percent identity for each full duplicated segment pair and for non-overlapping length-100 windows within TEs in those pairs. To identify windows with significantly lower identity than the background identity of the segment, we computed the binomial CDF and adjusted for multiple testing (due to multiple windows) by Bonferroni correction. Table 5 presents the proportion of TE pairs containing at least one low-identity window (*P*<0.001), and shows that discordant pairs are much more likely than concordant pairs to manifest this signal of recombination. Results are presented for L1 pairs and for Alu pairs broken out to the three types (AluJ, AluS, and AluY; in order from oldest to youngest[14]). Even among the most recombination-rich subfamilies (AluY), fewer than 30% of discordant pairs show a signal of having endured recombination. Though the precise percent of recombination is likely wrong due to the simplified model, the test highlights the much higher apparent recombination in discordantly-annotated pairs, suggesting that recombination may be the cause of some observed discordance. Note that this method is not expected to find all instances of recombination (see Discussion).

**Table 5:** Discordant TE pairs show higher rates of apparent recombination

	% of pairs w/ evidence of recombination		Mean % id to consensus
	Concordant	Discordant	
L1	1.3%	2.8%	79.9%
AluJ	1.6%	5.8%	87.4%
AluS	3.7%	19.0%	93.1%
AluY	5.1%	27.6%	96.2%

For each segmental duplicate TE pair, average segment percent identity was computed over the length of the segment. Then percent identity was computed for non-overlapping length-100 windows for each TE pair. We identified TEs containing windows with significantly reduced identity relative to the containing segmental duplication (*P*<0.001, Bonferonni correction applied to account for possibly-multiple windows per TE). We quantified the observed rates for Alu and L1 subfamilies, computing apparent recombination in both discordant and concordant pairs

### Subfamily annotation confidence can be quantified, reflects reliability

When a TE family is represented by several highly similar subfamily sequences, an instance of the family belonging properly to one subfamily may align with high score to many or all of the subfamily elements. An annotation pipeline must pick from among these high-scoring candidate annotations. When scores of these competing annotations are similar, the standard annotation-based-on-highest-score strategy overstates confidence. An extreme example is in the case where two alignments supporting competing annotation have the same score (often because the library sequences are identical over the aligned region): confidence in assigning the sequence to one subfamily or the other should be no greater than 50%, since either one is an equally good option.

**Fig. 2:**
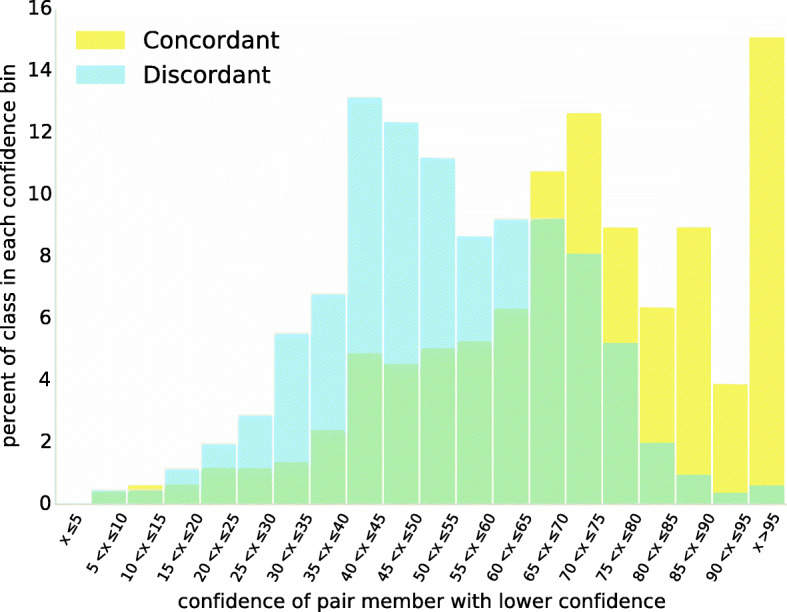
Annotation confidence tends to be lower in discordant TE pairs. Consider an Alu pair: the confidence can be computed for the best-supported annotation of both elements of the pair, and the smaller of these two confidence values may be thought of as a measure of the confidence that both halves have been correctly annotated. We computed this smallest-best-confidence value for each pair from the segmental duplicate dataset, dividing data into discordant pairs (in which best-supported annotations differ) and concordant pairs (in which best-supported annotations agree). Pairs were binned according to smallest-best-confidence, so that each bin represents the fraction of all discordant- or concordant-pairs with that bin’s smallest-best-confidence value. The left shift in discordant pairs indicates that, among discordantly-annotated elements, confidence is generally lower that both annotations are correct

Using a calculation of annotation confidence based on the ensemble of competing annotations (see Methods), we find that discordant Alu annotations in the segmental duplication dataset show significantly lower confidence in at least one of the pair of annotations than is seen in concordant annotations (Fig. 2; *P*-value 9.4∗10^−116^ according to the Kolmogorov-Smirnov test). For each pair, the preferred annotation was identified for both elements, and the element with the lower confidence among the pair was selected. Among concordant pairs, the median of these less-confident elements showed 73.4% confidence, while the median for discordant pairs was 52.8%. These results show that discordant pairs are likely to include at least one element with uncertain annotation.

## Discussion

Our study highlights problems with reliability in subfamily annotation, but we have certainly not explored all possible annotation schemes or databases. We have also specifically focused on a sequence-based library, rather than annotation with profile hidden Markov models (HMMs) as with Dfam [15] and nhmmer [16]. This was done in order to avoid possible confounding effects due to stochastic issues arising during sequence alignment and model construction (anecdotally, subfamily discordance appears to be at least as prevalent in Dfam-based annotation). Even so, the observed level of annotation discordance motivates our recommendation that TE researchers should be cautious in developing subfamily libraries, and in their application to genome annotation. Specifically: (i) when developing subfamily libraries, we recommend that TE researchers use measures of reliability to decide how aggressively to split families into subfamilies, and (ii) when using subfamily libraries for annotation, software pipelines should be adjusted to provide a measure of annotation confidence, and tools used in downstream analyses should account for this variability in annotation confidence.

The causes of the high levels of unreliability are multi-faceted. We have explored two possible causes, in the form of point mutation and homologous recombination. Previous work [17] has raised concern that the subfamilies incorporated into standard repeat libraries do not accurately reflect the complex histories of the families. This likely also contributes some of the inconsistent subfamily annotation. Consider, for example, the case in which a master element produces a number of instances, but a corresponding subfamily is not included in the library; in this case, all those instances will be annotated as belonging to some subfamily that arose in time *close* to the true subfamily. If there are two *close* subfamilies, then two duplicated copies may be assigned to those subfamilies based on random non-discriminatory mutations. This would be expected to produce reduced-confidence and possibly-discordant annotations, so may explain some of our observed discordance.

We have described a simple method for identifying possible instances of recombination, but caution that this method should not be used to quantify recombination rates - it is primarily useful as a method for showing differences in apparent recombination rates in discordant and concordant pairs. Recombination is most likely to occur when the donor sequence is highly similar to the acceptor sequence [18], so that many cases of recombination are expected to leave little trace in the form of sequence identity shifts. We also highlight that our analysis does not include recombination events that lead to the deletion of a TE instance, as such events would by definition not leave a pair that could be discordantly annotated. For a similar reason, our analysis only captures recombination events involving two break points surrounding a single region within the TE, since a recombination with a single breakpoint would split a segmental duplicate region in half, and thus escape our analysis pipeline.

Finally: our analysis has focused on subfamilies designed to represent the biology of TE instances (age, species-specificity, evolutionary history, etc.), specifically on human Alu and L1 subfamilies. Some subfamilies (such as MIR and L2 subfamilies) in databases like RepBase are used simply to increase annotation coverage by representing different regions of sequence space. In these cases, subfamily assignments should also be used with caution, but for the more mundane reason that those subfamilies only communicate something about the search mechanism, not about the biology of the sequence element.

## Methods

### Discordant annotation of TEs in segmental duplications

Our segmental duplicate analysis incorporated two independent databases: (1) segmental duplications from [8, 19] and (2) transposable elements from RepeatMasker [1]. The database of segmental duplications consists of 25,800 pairwise alignments. All duplications are greater than 1,000 base pairs long and at least 90% identical, so the duplications probably occurred in the last 40 million years [19]. RepeatMasker results were from RepeatMasker open-4.0.5, using RepeatMasker Repbase Library 20140131 [2] (at the time of publication, this is the most up-to-date release on the RepeatMasker website). In the entire genome, RepeatMasker identified 5,467,457 TE remnants classified into 1183 different subfamilies. Because segmental duplicates were identified in human genome hg19, RepeatMasker results were also downloaded for hg19.

TE duplicate pairs were identified based on the sequence alignment captured in the segmental duplication data from [19], which include segment context beyond the length of paired TE instances. To avoid TEs within segments with complicated histories, we restricted our analysis to segmental duplicates with only two copies, and found on canonical chromosomes 1-22, X, and Y. We took several steps to filter TE pairs that might be the result of independent insertions and so are not biological replicates. We considered only TE pairs in which both copies were longer than 50 nucleotides long, and at least 80% of the length of each copy was covered by the other, to avoid cases in which one copy is differently classified based solely on being much shorter than the other. Further, we retained only TE pairs in which the pair are related in one contiguous alignment, to avoid cases of nuanced annotation due to, for example, a large insertion or deletion in one element of the pair following duplication. Finally, we ignored pairs in which at least one element was labeled ambiguously, with no specific subfamily (i.e. *Alu*). There were 16,962 instances of these straightforward TE duplications. When two aligned TEs were assigned by RepeatMasker to different subfamilies, we labeled the TE pair as discordantly annotated.

### Comparison of TEs in human and chimp

We analyzed TEs annotated by RepeatMasker in the human genome (hg38) and the chimpanzee (*Pan troglodytes*) genome (panTro4). To find homologous pairs, we downloaded BED files of the annotations from the UCSC Table Browser [20] and used the liftOver tool [9] (downloaded on 14 April 2017) to convert the coordinates of TEs in the human genome into coordinates in the chimpanzee genome. We then used BEDTools [21] to find overlapping TEs and identified discordant annotation as before.

### Subfamily conversion due to point mutations

For each subfamily, we estimate the probability that a nucleotide remains unchanged after a TE instance is inserted in the genome, *P*(*A*_*i*_), as the mean percent identity between the subfamily consensus and individual TE instances annotated by RepeatMasker. The probability that a specific site will change from the diagnostic (subfamily-specific) nucleotide is then (1−*P*(*A*_*i*_)). Assuming uniform chance of mutating to each of the other three nucleotides, the probability that a diagnostic site for subfamily *i* will change to the value associated with another subfamily *j* is *P*(*B*_*ij*_)=(1−*P*(*A*_*i*_))/3; the remaining probability *P*(*O*_*ij*_)=2(1−*P*(*A*_*i*_))/3 is that chance that the diagnostic site will mutate away from the diagnostic value for subfamily *i* to a nucleotide other than the one that is diagnostic for subfamily *j*. Note that these mutation probabilities are since-insertion, not since-duplication, because the inserted element may accumulate some mutations suggestive of subfamily *j* prior to duplication.

Consider an Alu instance *S* belonging to subfamily *i*, and suppose that at the moment of insertion (at some point prior to duplication), it agreed with the consensus for *i* at all *n* diagnostic sites that differentiate the consensus of *i* from the consensus of subfamily *j*. One history that would cause one copy of *S* to be identified as belonging to subfamily *j* is for at least *n*/2 of those diagnostic sites to mutate to agree with subfamily *j*, and that no diagnostic sites mutate to some other value that disagrees with both *i* and *j*. The probability of this occurring is the product of (i) the probability of no mutations of a diagnostic site to an *other* value, and (ii) the probability that fewer than *n*/2 diagnostic sites do not change from the value for *i* (the cumulative probability from the Binomial distribution):

1 $$ \begin{aligned} P(S:i\rightarrow{}j, other=0) &= (1-P(O_{ij}))^{n}\\& \cdot B\left(\left\lfloor{\frac{n-1}{2}}\right\rfloor, n, P(c:i \rightarrow j)\right) \end{aligned}  $$

where the probability of a diagnostic site not changing from *i* to *j*, given that it also did not change to an *other* value is:

2 $$ P(c:i \rightarrow j) = \frac{p(A_{i})}{(1-P(O_{ij}))} = \frac{3p(A_{i})}{1+2p(A_{i})}  $$

and the Binomial CDF is:

3 $$ B(x, n, p) = \sum_{i=0}^{\lfloor x \rfloor} {n\choose i}p^{i}(1-p)^{n-i}  $$

More generally, if some number *k* of the *i*-diagnostic sites mutate to a non-informative *other* state, then only (*n*−*k*)/2 sites need to change to agree with *j*, so that the overall probability of *S* being identified as belonging to *j* based on diagnostic sites is:

4 $$ \begin{aligned} P(S:i\rightarrow{}j) &= \sum_{k=0}^{n-1} {n \choose k} (1-P(O_{ij}))^{n-k} P(O_{ij})^{k}\\ &\cdot B\left(\left\lfloor{\frac{n-k-1}{2}}\right\rfloor, n-k, P(c:i \rightarrow j) \right) \end{aligned}  $$

Equation 4 was used to compute the probability of converting an instance of subfamily *i* to be recognized as belonging to subfamily *j*, for each pair of subfamilies. Then for each subfamily, a weighted average of these probabilities was computed for each type (J,S,Y), based on the observed frequency of each subfamily in the human genome (from http://repeatmasker.org).

### Computing subfamily annotation confidence

We compute a measure of confidence that the annotated sequence belongs to a subfamily *i* by leveraging the probabilistic underpinnings of alignment scores.

Suppose we have *Q*=*q*_1_,*q*_2_,...,*q*_*n*_ competing subfamily annotations of genomic sequence *t*. If we define *P*(*q*_*i*_|*t*) as the probability that the true label of *t* is *q*_*i*_, then the confidence that *q*_*i*_ is the correct label is

5 $$  \text{Conf}(q_{i}|t) = \frac{P(q_{i}|t)}{\sum_{j}{P(q_{j}|t)}}  $$

Assuming a uniform distribution over *Q*, *P*(*q*_*i*_|*t*)∝*P*(*t*|*q*_*i*_), so that

6 $$ \text{Conf}(q_{i}|t) = \frac{P(t|q_{i})} {\sum_{j}{P(t|q_{j})}}  $$

Under scoring matrices such as those used in RepeatMasker (based on cross_match [22], the score for aligning a pair of letters is based on a log odds ratio [23], where the ratio is “the probability of the two letters aligning if the sequences are homologous” vs “the probability of two letters aligning if the sequences are not homologous”. Typically, the real-valued log odds values are scaled by factor *λ* then rounded to the nearest integer value:

7 $$ \text{score}(a,b) = \text{int} \left(\lambda \log \frac{P(a,b)}{P(a) P(b)} \right)  $$

In an alignment with no insertions, the overall alignment score corresponds to a scaled log of the ratio of the probability of observing *t* if it is homologous to *q*_*i*_ vs the probability of observing *t* under a random (non-homology) model:

8 $$  \text{score}(t,q_{i}) = \lambda \cdot \log \frac{P(t|q_{i})}{P(t|R)}  $$

Though typically these scores are integer-rounded, and alignment gap penalties are ad hoc (read: not derived from probabilities), we accept a simplifying approximation that they map to feasible probabilities [24], and utilize Eq. 5 in computing confidence values. This implies that

9 $$ P(t|q_{i}) = P(t|R) \cdot 2^{\text{score}(t,q_{i}) / \lambda}  $$

and after straightforward algebraic manipulation following substitution into Eq. 5,

10 $$  \text{Conf}(q_{i}|t) = \frac{2^{(\text{score}(t,q_{i}) / \lambda)}} {\sum_{j}{2^{(\text{score}(t,q_{j}) / \lambda)}}}  $$

This approach is admittedly simplistic, in that it assumes that all competing sequence alignments cover the same genomic range (what we’ve called *t*). Even so, it allows us to inspect the relationship between confidence in subfamily annotation and the risk of discordance due to accumulation of point mutations.

Alignments used for annotation with RepeatMasker are produced using cross_match with custom scoring matrices based on regional GC content. For each segmental duplicate Alu pair (*t*_1_,*t*_2_), we first infer the *λ* value for the region-specific scoring matrix using the esl_scorematrix executable available via special compilation of the Easel sequence analysis library (http://bioeasel.org, implementing the method of [23]). Using this and alignment scores, we used Eq. 10 to compute estimates of the confidence for the best-scoring annotation for both *t*_1_ and *t*_2_, then captured the lower confidence value for that pair: $m = \min (\text {Conf}(\hat {q}|t_{1}), \text {Conf}(\hat {q}|t_{2})$. The distributions of the smallest-maximum-confidence values (Fig. 2) were significantly different under the Kolmogorov-Smirnov test.

### Computing recombination estimates

To estimate the rate of recombination in the segmental duplicate TE pairs, we compared the identity of pairs of TEs to the identity of the segments containing them. Each segmental duplication is described by an alignment of the sequence of the original segment and the sequence of the duplicated segment; these can be many kilobases in length, and can contain multiple TE instances. For each segmental duplication alignment, we computed the percent identity as the number of columns containing identical nucleotides in both sequence, divided by the number of non-gap columns in the alignment. Then for each TE pair *p* identified via the previously-described filtering process, we split the alignment of *p* into non-overlapping windows of 100 non-gap columns, starting at the first aligned position. We counted the number of identical columns *c* among these 100, and computed the binomial CDF (the probabilitiy of observing *c* or fewer identical columns out of 100, given the overall percent identity of the entire segmental duplication alignment). For each TE pair, we captured the smallest identity count among all windows, then subjected the corresponding binomial CDF value to Bonferroni correction to account for possibly-multiple windows. We reported TE pairs with *P*<0.001, for both discordant and concordant pairs. We selected windows of length 100 because gene conversion events are typically at least 50 base pairs long [12]. Because we captured non-overlapping windows, the final (*n* mod 100) columns of an *n* column alignment are not used for the recombination estimate; this likely results in an under-estimate of recombination frequency.

## Data Availability

The datasets analyzed and generated for this study, along with the scripts used for analysis, are available at http://wheelerlab.org/publications/2020-CareyPatterson/CareyPatterson_suppl.tar.gz.
